# Interplay between Chaperones and Protein Disorder Promotes the Evolution of Protein Networks

**DOI:** 10.1371/journal.pcbi.1003674

**Published:** 2014-06-26

**Authors:** Sebastian Pechmann, Judith Frydman

**Affiliations:** Department of Biology, Stanford University, Stanford, California, United States of America; University of California San Diego, United States of America

## Abstract

Evolution is driven by mutations, which lead to new protein functions but come at a cost to protein stability. Non-conservative substitutions are of interest in this regard because they may most profoundly affect both function and stability. Accordingly, organisms must balance the benefit of accepting advantageous substitutions with the possible cost of deleterious effects on protein folding and stability. We here examine factors that systematically promote non-conservative mutations at the proteome level. Intrinsically disordered regions in proteins play pivotal roles in protein interactions, but many questions regarding their evolution remain unanswered. Similarly, whether and how molecular chaperones, which have been shown to buffer destabilizing mutations in individual proteins, generally provide robustness during proteome evolution remains unclear. To this end, we introduce an evolutionary parameter **λ** that directly estimates the rate of non-conservative substitutions. Our analysis of **λ** in *Escherichia coli*, *Saccharomyces cerevisiae*, and *Homo sapiens* sequences reveals how co- and post-translationally acting chaperones differentially promote non-conservative substitutions in their substrates, likely through buffering of their destabilizing effects. We further find that **λ** serves well to quantify the evolution of intrinsically disordered proteins even though the unstructured, thus generally variable regions in proteins are often flanked by very conserved sequences. Crucially, we show that both intrinsically disordered proteins and highly re-wired proteins in protein interaction networks, which have evolved new interactions and functions, exhibit a higher **λ** at the expense of enhanced chaperone assistance. Our findings thus highlight an intricate interplay of molecular chaperones and protein disorder in the evolvability of protein networks. Our results illuminate the role of chaperones in enabling protein evolution, and underline the importance of the cellular context and integrated approaches for understanding proteome evolution. We feel that the development of **λ** may be a valuable addition to the toolbox applied to understand the molecular basis of evolution.

## Author Summary

Evolutionary innovation through mutation is important for adaptation, thus ultimately survival of all species. Proteins, the main actors of cellular life, are generally only marginally stable in the cell and sensitive to mutations. This raises the question how the emergence of new functions is balanced with the destabilizing effect of mutations. Here, we incorporate biophysical knowledge on the stability effect of mutations into an extended model of protein evolution to better understand at the proteome level factors that promote non-conservative, more likely destabilizing mutations. Our analyses reveal a central role of molecular chaperones, specialized quality control enzymes that are known to promote protein evolvability by buffering the destabilizing effect of mutations in individual substrates. We find that chaperones support non-conservative mutations at the proteome level both directly, and through stabilizing intrinsically disordered proteins. We demonstrate how the main co- and post-translationally acting chaperones distinctly promote the same non-conservative mutations that characterize the re-wiring of protein interactions. Our results thus suggest how energetically costly protein quality control pathways can systematically promote the evolution of protein networks, and highlight the importance of considering the cellular context for understanding protein and proteome evolution.

## Introduction

Protein evolution is central to adaptation and ultimately survival of all species [1]. Proteins evolve through mutation and selection, and given the marginal stability of their native state and the sensitivity of protein structure to mutation [2], major questions arise concerning how the emergence of new functions is balanced with the destabilizing effect of mutations [3], [4]. Importantly, the cell has an elaborate quality control machinery to target destabilized and misfolded proteins for either refolding or degradation [5]. The interplay between acceptance and selection of mutations and cellular protein quality control is a relatively unexplored factor in protein evolution. It is proposed that cellular factors, such as molecular chaperones, can stabilize mutant proteins [6], thereby providing extrinsic robustness against mutations [7] [8]. However, a detailed understanding of the role of chaperones in proteome evolution is currently lacking.

The evolution of protein coding genes is commonly described by the rates of non-synonymous substitutions, or amino acid changes, *dN*, and synonymous or silent substitutions, *dS*. Their rate ratio **ω** = *dN/dS* is widely used to detect the strength of positive or purifying selection on protein sequences [9]. These analyses have shaped our understanding of protein evolution, demonstrating for instance that sequences evolve at markedly varying rates [10], [11], [12]. Thus, highly expressed proteins evolve at a lower rate of amino acid changes *dN* [13], likely because the cost of deleterious mutations leading to misfolding and aggregation increases with protein abundance. Consequently, highly expressed proteins are subject to stronger purifying selection to maintain high translational fidelity [14], stability [15], and solubility [16].

Despite the usefulness of **ω** = *dN/dS* to understand broad evolutionary selective pressures, it is more limited in quantifying *how* proteins evolve. Recent efforts to carefully integrate structural information have revealed distinct modes of evolution in buried and exposed residues [17], [18], [19], [20]. However, detailed structural information is only available for a limited set of highly expressed and soluble proteins. Generally, biophysical and genetic analyses indicate that conservative (C) substitutions between more similar amino acids, e.g. from *Leu* to *Ile*, often have very little effect on a protein, whereas a non-conservative (NC) mutation is more likely to affect protein stability and function [21]. By amalgamating all non-synonymous substitutions into one rate *dN*, **ω** does not discriminate between the disparate effects of different amino acid mutations on protein structure and function, which is important for gaining further insight into the forces that shape protein evolution. Importantly, proteins are only marginally stable [2] and soluble [22] in the cell, and protein stability is a major constraint on protein evolution [23], [24]. Indeed, mutations causing loss of protein stability and solubility are generally deleterious [25]. Furthermore, mutations that lead to new protein functions are often highly destabilizing [26], [27]. This renders protein evolvability a balancing act between accepting beneficial substitutions and providing sufficient robustness against their deleterious effects.

We hypothesized that incorporating the stability effect of mutations into an extended model of protein evolution may shed light onto how cellular factors influence the evolution of protein coding sequences. To this end, we developed an evolutionary parameter, **λ**, that directly infers the rate ratio of NC and C substitutions. Our rationale for **λ** is the demonstration in many experimental systems and biophysical analyses that NC mutations are generally more likely destabilizing than C mutations [21], thus providing a good approximation for the stability effect of mutations given the constraints imposed by sequence-based evolutionary models (*see* *Methods*). Supporting this assumption, we find that, unlike **ω**, **λ** can capture known instances of rapid and distinct evolution, namely, those of intrinsically disordered proteins and those acting as re-wired nodes in protein-protein interaction networks. In these cases, NC mutations appear to drive new protein interactions and thus novel functions. Importantly, analyses of **λ** in the proteomes of *E. coli*, *S. cerevisiae*, and *H. sapiens* revealed higher **λ** values in chaperone substrates, suggesting that chaperones globally promote the acceptance of NC substitutions. Taken together, our analysis suggests that the mutations that are both on average more destabilizing and more likely leading to novel protein interactions are also preferentially found in chaperone substrates. These findings obtained at a systems-level for three different organisms agree with individual biochemical studies indicating a capacitor role of specific chaperones in evolution [6], [7], [8]. Lastly, our analysis, showing that the re-wiring of protein interactions at the proteome level is linked to energetically costly protein quality control, suggests a cost-benefit trade-off in the evolvability of protein networks.

## Results

### λ: An evolutionary parameter that considers the stability effect of mutations

**Figure 1:**
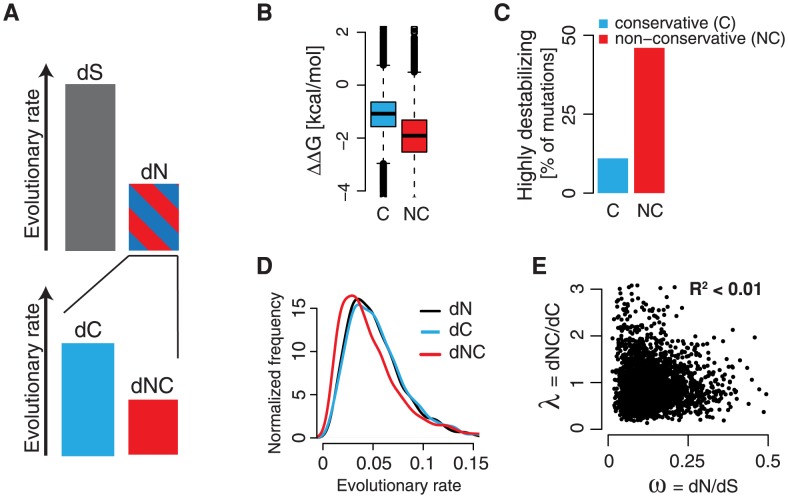
An extended model of protein evolution incorporates the stability effect of mutations. **A** Conventionally, protein evolution is described by the rates of non-synonymous substitutions *dN*, and synonymous substitutions *dS*, and their rate ratio **ω** = *dN/dS*. To accommodate the disparate effect of different amino acid changes on protein stability, the rate of non-synonymous substitutions *dN* is split into the rates of conservative substitutions *dC*, and non-conservative substitutions *dNC*. **B** Predicted stability effects of conservative (C) and non-conservative (NC) mutations. NC mutations are more likely destabilizing than C mutations. **C** NC mutations are much more likely highly destabilizing (**ΔΔ**G<−2 kcal/mol) than C mutations. **D** The evolutionary rate *dNC* is on average lower than *dC* and *dN*, suggesting that NC substitutions are generally under stronger purifying selection. **E** The evolutionary rate ratios **ω** = *dN/dS* and **λ** = *dNC/dC* are not correlated, thus independent parameters that contribute orthogonal insights.

The 20 naturally occurring amino acids differ widely in their physicochemical properties. Consequentially, not all amino acid substitutions equally affect protein structure and function. More differentiated descriptions of protein evolution beyond the rate of non-synonymous substitutions *dN* are needed to better describe the forces shaping protein evolution. Initial classifications into “conservative” substitutions between more similar, and “radical” substitutions between more dissimilar amino acids focused for instance on charge and polarity [28], [29]. Recent experimental and theoretical work underlined the pivotal role of protein stability as a major constraint on protein evolution [23], [24]. We hypothesized that incorporating the stability effect of mutations into the computation of evolutionary rates may provide more nuanced insights into the evolution of protein coding sequences (Figure 1A). To test this assumption, we derived a classification into conservative (C) and non-conservative (NC) substitutions hat reflects this important biophysical constraint. Because the effect of mutations on protein stability *in vivo* is highly contextual, depending on the local and global protein fold, and is not easily measurable in high-throughput, we predicted the stability effect of a large set of mutations for the *S. cerevisiae* proteome *in silico* [30]. We found very clear differences in the distributions of the predicted stability changes **ΔΔ**G for different amino acids pairs (Figure S1A). Computational models of protein evolution that are based on sequence information alone require a strict classification of mutations into either C or NC substitutions. We computed for each amino acid pair that is separated by one nucleotide substitution in the genetic code the fraction of mutations that was predicted highly destabilizing (**ΔΔ**G<−2 kcal/mol). Because a hard classification into C or NC substitution is of necessity an approximation that neglects the detailed structural context of mutations for that particular protein, we sought to define only the most frequently destabilizing mutations as NC (Figure S1B). Because mutations that were predicted highly destabilizing in more than 20% of the cases clearly separate from the rest (Figure S1B), we choose 20% as the best threshold. A more stringent threshold reduces the number of NC mutations (Figure S1C,D), but increases the difference in predicted stabilities between C and NC substitutions (Figure S1E). Too few NC mutations make it difficult to estimate evolutionary parameters due to diminishing sample sizes. We thus classified all amino acid changes into NC substitutions if they were predicted to be highly destabilizing more than 20% of the time, and as C otherwise (*see* *Methods*). We further validated that this classification is in agreement with the established Blosum62 amino acid substitution matrix in that no NC mutation has a positive Blosum62 score. In our classification, NC substitutions are significantly more destabilizing than C substitutions (Wilcoxon rank-sum test: p<<10^−16^, standardized mean difference SMD = 0.96, Figure 1B). Indeed, less than 10% of C mutations, but almost 50% of NC mutations were predicted to be highly destabilizing (**ΔΔ**G<−2 kcal/mol, Figure 1C). One caveat of our approach is that, while the grouping into classes of mutations reduces their dependence on the accuracy of individual predictions, our approach is prone to systematic biases in the stability predictions. We therefore compared our classification to established predictors of the phenotypic consequences of mutations, namely PolyPhen [31] and SNPs3D [32], as well as fitness values for mutations from deep sequencing data [33]. Our analyses all support a significantly stronger negative phenotypic or fitness effect of NC substitutions compared to C substitutions (Figure S2A–C), but simultaneously highlight the potential for novel data to improve these classifications. Thus, while our classification procedure is *ad hoc* and only represents one of several possible avenues to predict and integrate the stability effect of mutations, our approach appears to be useful for comparing NC and C as a proxy for incorporating the stability effect of mutations into an extended evolutionary model.

We next implemented a Markov model of codon substitutions that directly estimates the rates of conservative substitutions *dC*, and non-conservative substitutions *dNC* in complement to the established rates *dN* and *dS* (Figure 1A, S3A–D). As expected, *dNC* is on average lower than *dC*, reflecting stronger purifying selection on substitutions that are more likely destabilizing (Figure 1D). In analogy to the evolutionary rate ratio **ω** = *dN/dS*, we defined the evolutionary parameter **λ** = *dNC/dC*. Whereas **ω** describes the relative rate of all selected mutations that result in an amino acid change, the new parameter **λ** informs on the relative partitioning of substitutions that are more or less likely destabilizing. Importantly, **ω** and **λ** are not correlated, thus independently describe orthogonal aspects of protein sequence evolution (Figure 1E). Furthermore, because **λ** is not correlated to expression levels (Figure S3E,F), this new parameter can shed light on factors that promote non-conservative substitutions even in highly expressed proteins that have overall low rates of amino acid changes.

### λ describes the evolution of intrinsically disordered proteins

An interesting correlate and test case for our new evolutionary parameter **λ** is presented by the analysis of intrinsically disordered proteins (IDPs). Disordered regions in proteins maintain a distinct composition of polar and charged amino acids, and IDPs are under tight cellular regulation to prevent their aggregation [34] [35]. In turn, intrinsic disorder is known to perform functionally important roles in molecular recognition [36], and thus cellular interaction networks [37]. Protein disorder also plays a unique role in protein evolution. Disordered regions themselves evolve on average more rapidly, attributed in part to the lack of structural constraints [38], [39]. Moreover, disordered regions have been shown to exhibit distinctly different amino acid substitution patterns with a much higher frequency of NC substitutions [40]. We thus next sought to evaluate the power of **λ** based on the more general classification of NC and C defined above, to describe the evolution of IDPs.

**Figure 2:**
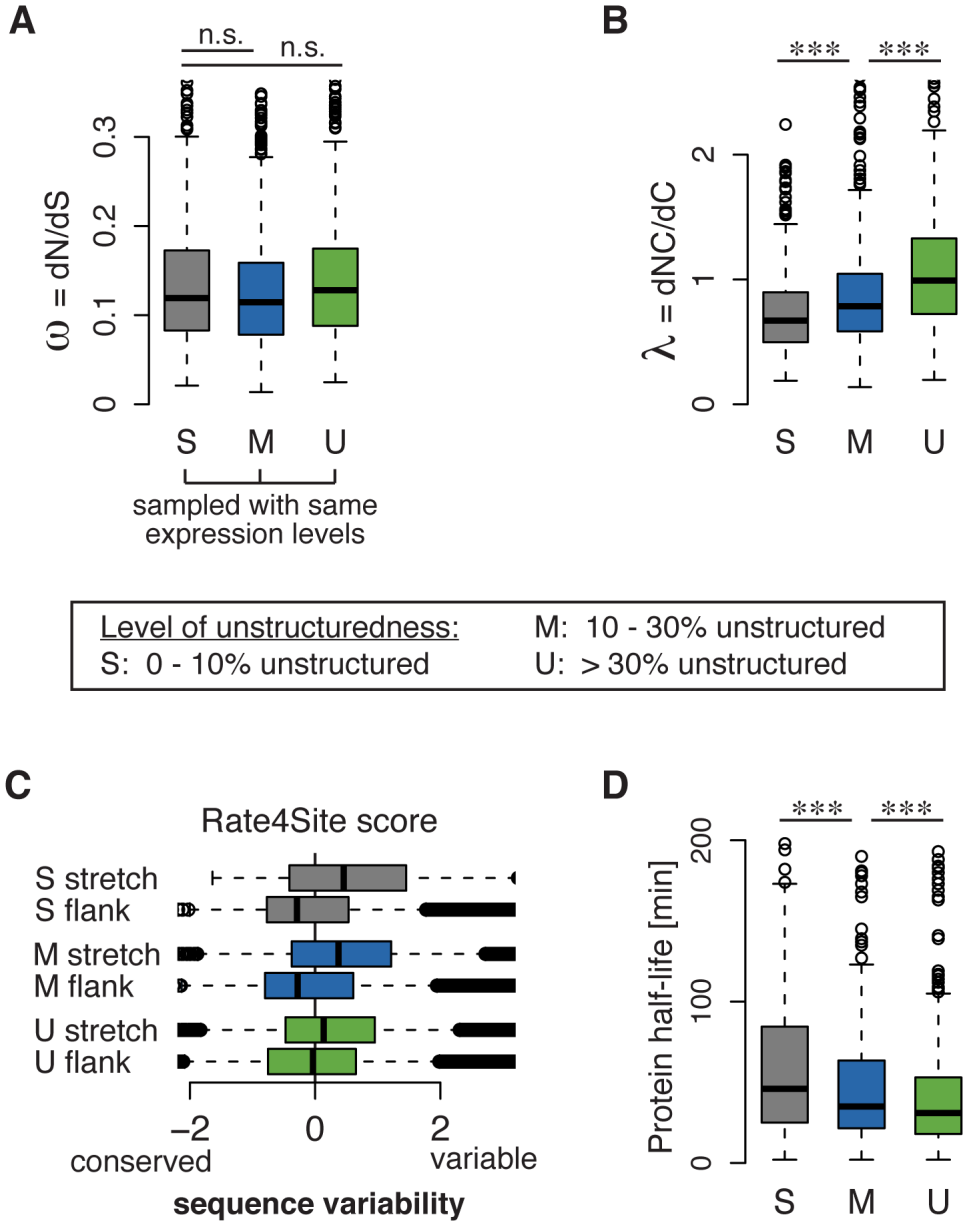
λ describes the evolution of intrinsically disordered proteins. Proteins are classified according to their percentage of residues that is predicted to be disordered (S: <10%, M: 10–30%, U: >30%). **A** The evolutionary rate ratio **ω** is largely independent of the level of protein structuredness for proteins sampled with similar levels of expression. **B** The evolutionary rate ratio **λ** increases with increasing protein disorder. **C** Comparison of the local sequence variability and conservation of the unstructured regions (stretch) to the immediately adjacent structured flanking regions (flank) with the Rate4Site algorithm [41] indicates compensatory selective pressure in the flanking regions. **D** Protein half-lives on average decrease with increasing protein disorder. Significance levels are indicated as n.s. (not significant), and *** (p<0.001).

We divided the *S. cerevisiae* proteome into three classes based on the percentages of residues that were predicted to be disorder, as described in [35]: S (“highly structured”, 0–10% disordered), M (“moderately unstructured”, 10–30% disordered), and U (“highly unstructured”, >30% disordered). When controlled for expression levels, which appear as the strongest determinant of the evolutionary rate ratio **ω**, we found that **ω** is not well suited to detect differences in the evolution of proteins of different levels of structuredness (Wilcoxon rank-sum test; S vs. M: p = 0.15, SMD = 0.05; S vs. U: p = 0.19, SMD = 0.11; M vs. U: p = 0.007, SMD = 0.14; Figure 2A, S4A). In contrast, using **λ**, we found that structured proteins evolve at significantly lower **λ** than moderately unstructured proteins (Wilcoxon rank-sum test: p<10^−6^, SMD = 0.34, Figure 2B). Unstructured proteins evolve at an even higher **λ** than moderately unstructured proteins (Wilcoxon rank-sum test: p<10^−13^, SMD = 0.43, Figure 2B). Importantly, both **ω** and **λ** are computed for the full protein coding sequences. While **ω** is not correlated with disorder, **λ** is strongly correlated, and increases with the disordered content in proteins (R_S_ = 0.38, Figure S4B,C). How does **ω** not depict the known accelerated evolution of disordered regions when controlled for expression levels? To understand this discrepancy, we computed the Rate4Site scores [41] of the local sequence variability of disordered regions and their adjacent structured flanking regions. Our analysis revealed that, while unstructured stretches are indeed more variable, their structured flanking regions in contrast are very conserved (Figure 2C). This observation suggests systematic compensatory selection in the flanking regions, likely to facilitate the variability of disordered regions [42]. This combination of variable and conserved regions often results in comparable rates of overall non-synonymous substitutions between structured and more disordered proteins, thereby obscuring the more rapid evolution of the disordered regions in IDPs. As a result, while **ω** is limited in its power to characterize the evolution of proteins with disordered regions, **λ** identifies the much higher rate of NC mutations linked to intrinsic disorder. This analysis indicates, even in the general parsimonious classification used here, that **λ** can quantify and contribute additional insights into the evolution of IDPs in complement to **ω**. Although NC mutations may not “destabilize” intrinsically disordered regions, they can still perturb protein dynamics, protein interactions and homeostasis. In response, intrinsically disordered proteins are under tight regulation and turned-over more rapidly (Figure 2D) [35]. This is energetically costly, but likely prevents their aggregation [43], and non-specific interactions [44]. The documented evolutionary benefit of intrinsically disordered regions thus comes at the expense of selective protein quality control. Through an integrated analysis, we show in the following the importance of **λ** to decipher this trade-off.

### λ is linked to the re-wiring of protein interaction networks

We next used **λ** to analyze the factors involved in the re-wiring of protein-protein interactions (PPIs). Evolving a novel protein interaction has been suggested to present one of the easiest routes to acquiring a novel protein function [45], and PPIs are re-wired substantially over the course of evolution [46]. Indeed, a single mutation may suffice for the design of novel protein interactions [47] [48]. Accordingly, generally only very few mutations are predicted to separate protein surfaces from interaction interfaces [49]. We thus hypothesized that **λ** might characterize the evolvability of protein interactions.

**Figure 3:**
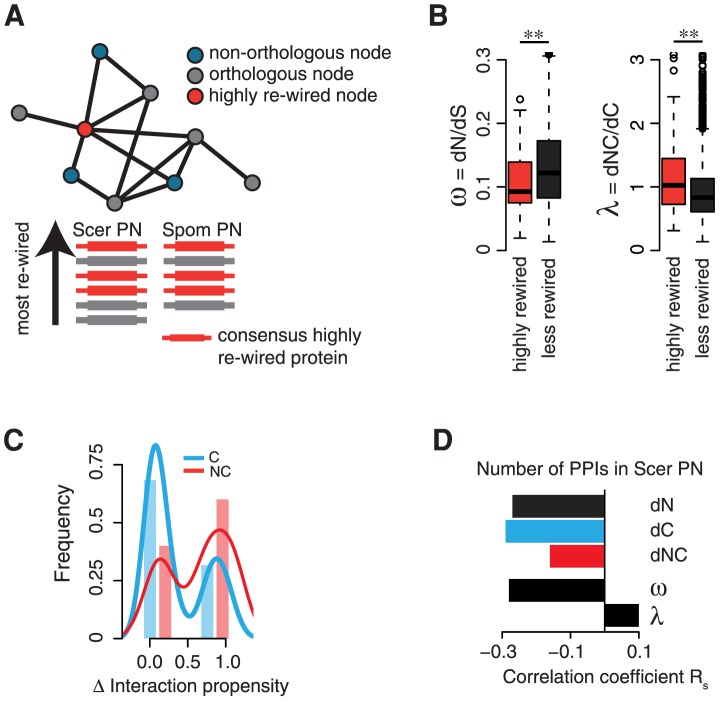
λ describes the re-wiring of protein-protein interactions. **A** The most highly re-wired proteins are derived from a consensus ranking of nodes with the most non-orthologous neighbors in the protein interactions networks (PN) of *S. cerevisiae* (Scer) and *S. pombe* (Spom). **B** Highly re-wired proteins evolve at lower **ω**, thus are under stronger purifying selective pressure, but evolve at clearly higher **λ** compared to less rewired proteins. **C** NC mutations are much more likely to comprise changes between amino acids with very different interaction propensities than C mutations. Shown are the distributions of the changes in interaction propensity of NC and C mutations (lines), and a simpler binary classification into high and low differential interaction propensities (bars, more transparent in the background). **D** The number of protein-protein interactions (PPIs) negatively correlates more strongly with *dC* than *dNC*, and only weakly with **λ**, suggesting that **λ** is not systematically biased by high connectivity in protein networks. Statistical significance is indicated as ** (p<0.01).

To assess the relationship between **λ** and the evolution of novel PPIs, we extracted the consensus most highly re-wired proteins from the protein networks (PN) of *S. cerevisiae* and *S. pombe* (Figure 3A, *see* *Methods*) [50]. We found the most highly re-wired proteins to be highly connected and evolving at clearly lower **ω** than the less re-wired proteins (Wilcoxon rank-sum test: p = 0.004, SMD = 0.38; Figure 3B). Highly connected proteins are important and functionally constrained hubs in PNs, often highly expressed, and thus evolving more slowly when described by **ω** [51]. Strikingly, the highly re-wired proteins exhibit significantly higher **λ** (Wilcoxon rank-sum test: p = 0.002, SMD = 0.57; Figure 3B). Thus, despite an overall high degree of conservation, i.e. lower rates of overall amino acid changes, the highly re-wired proteins exhibit elevated relative rates of NC substitutions, as expected when acquiring or remodeling interaction partners. Indeed, the link between NC mutations and the evolution of novel protein interactions is supported by a recent scale of amino acid interaction propensities derived from a careful analysis of curated protein complexes [52]. NC substitutions are much more likely to comprise changes between amino acids with very different interaction propensities (Fisher's exact test: p = 0.0002, Figure 3C), underlining that NC mutations more profoundly change the interaction potential of a protein. Importantly, the connectivity in the *S. cerevisiae* PN negatively correlates much more strongly with *dC* than *dNC*, (Figure 3D), and only very weakly with **λ**. Thus, the plasticity to accept NC substitutions is not constrained by network connectivity. We conclude that **λ** can shed light onto the evolution of protein interactions within cellular protein networks.

### Weak chaperone dependence promotes non-conservative mutations

We next used **λ** to better understand the cellular strategies that regulate NC substitutions. Besides loss-of-function mutations, organismal fitness is most impaired by the accumulation of destabilized, misfolded proteins leading to cytotoxic protein aggregates [5]. Because a higher **λ** indicates a higher relative rate of NC mutations, we asked whether cellular factors confer robustness against their potentially destabilizing effects. Chaperones, which promote folding and prevent aggregation [5] [53], have long been proposed to buffer mutations and play important roles in protein evolution [54], [55], [56]. For instance, enhancing chaperone capacity through over-expression has been directly shown to promote enzyme evolvability [6]. Chaperones have been found to act both as genotypic and phenotypic capacitors [54], [55], [56]. However, if and how chaperones systematically influence genome and proteome evolution remains to be understood. In some studies, native chaperone substrates have been found to evolve, unexpectedly, more slowly as revealed by low **ω** [57]. This observation appears to contradict the idea that chaperones promote evolvability. However, chaperone clients are often highly expressed, and highly connected in protein interaction networks [58], and thus may be functionally more constrained. This could explain why they evolve more slowly when judged by the **ω** parameter [51]. An analysis of site-specific evolutionary rates has found higher local sequence variability in GroEL clients compared to non-clients [59]. We here next examined in detail whether **λ** can detect and quantify a role for chaperones in protein evolution at a global, proteome wide level.

**Figure 4:**
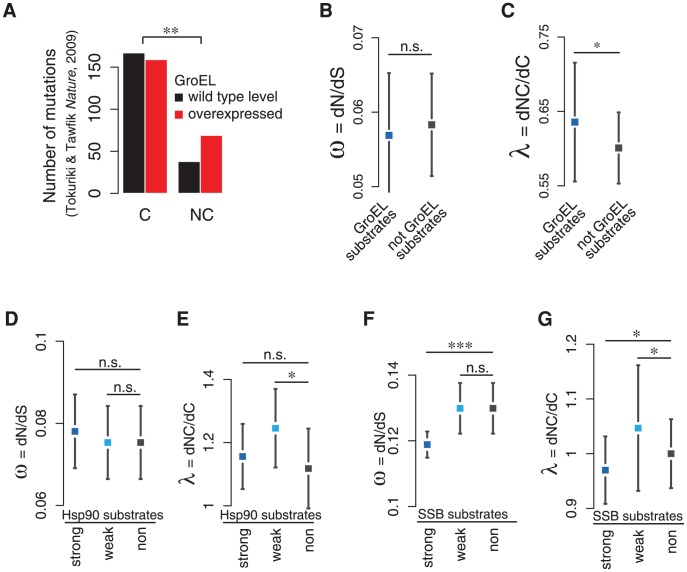
Weak chaperone dependence promotes NC substitutions. **A** The over-expression of GroEL in the directed evolution of enzymes reported in [6] promotes NC substitutions, thus validation the definition of NC mutations and **λ**. **B** Native substrates of the *E. coli* chaperonin GroEL (n = 58) do not evolve at higher **ω** than non-substrates (n = 216). Shown is the partial regression plot of the contribution of chaperone dependence on **ω**, together with 5% confidence intervals. The confidence intervals reflect the limited power of predicting a continuous variable, e.g. **ω**, from chaperone dependence alone. The significance of chaperone dependence is assessed by non-parametric regression analyses that explicitly include expression and disorder as confounding factors. **C** Native substrates of the *E. coli* chaperonin GroEL evolve at significantly higher **λ**. **D** Human kinases that are substrates of Hsp90 do not evolve at significantly higher **ω** than kinases that are not Hsp90 substrates when expression levels as confounding factor are included in the analysis. **E** Both strongly (n = 71) and weakly (n = 70) Hsp90 dependent kinases exhibit a significantly higher **λ** than non-substrates (n = 61). **F** Strong substrates of the yeast Hsp70 SSB (n = 648) evolve at lower **ω**, weak substrates (n = 310) at similar **ω** compared to non-substrates (n = 721). **G** Strong SSB substrates also evolve at lower **λ**, but weak substrates evolve at higher **λ**. Significance levels are indicated as n.s. (not significant), * (p<0.05), ** (p<0.01), and *** (p<0.001).

We first took advantage of data from a directed evolution experiment to test whether **λ** can reproduce a known role for chaperones in protein evolution. The chaperonin GroEL is essential in most prokaryotes, and assists the post-translational folding of topologically complex and aggregation-prone proteins [60]. Over-expression of GroEL in *E. coli* promoted enzyme evolution and acquisition of novel functions [6]. Our analysis of the mutations selected in the directed evolution experiments by Tawfik and co-workers indicated a significantly higher fraction of accepted NC substitutions for proteins that evolved at higher GroEL expression level, suggesting that the chaperone helps buffer NC mutations (Fisher's exact test: p = 0.005; Figure 4A). After validating our approach with this known and well–characterized example, we next extended our analysis to the evolution of known *in vivo* substrates of GroEL. Importantly, in the following section we systematically tested for an effect of chaperones on protein evolution by non-parametric regression analyses that estimate the contribution of the chaperone independently of other determinants such as levels of expression and disorder (*see* *Methods*). We found that **ω** cannot distinguish between GroEL substrates and proteins that do not interact with GroEL *in vivo* (p = 0.16, Figure 4B). In contrast, GroEL substrates evolve at significantly higher **λ** than non-substrates (p = 0.025, Figure 4C). Thus, while here in this integrated analysis that also considers potential confounding factors such as expression levels and disorder the established evolutionary rate ratio **ω** fails to detect any influence of the chaperone on protein evolution, **λ** indeed finds a higher rate of NC substitutions in the GroEL substrates (Figure 4C, S5A). Of note, bacterial proteomes have a low content of disorder compared to eukaryotes [61], [62] (Figure S5B), and almost all GroEL substrates are highly structured (Figure S5A).

In comparison to prokaryotic genomes, the role of chaperones in eukaryotes is less defined at a systems level. The heat-shock chaperone Hsp90 facilitates the maturation of many oligomeric complexes, as well as regulatory and signaling proteins, and also protects stress-denatured polypeptides [58] [63] (Figure S5C). A recent very elegant study identified a large set of kinases in *H. sapiens* as Hsp90 substrates, classified according to their strong and weak dependence [64]. Strong substrates more stringently depend on chaperone assistance for folding. Weak substrates in turn interact more sporadically and do not necessarily require chaperone assistance. Hsp90 was found to promote protein evolutionary rates in strong substrates when assessed by *dN* or **ω** [64]. However, this analysis did not take into account expression levels [64], which are known to strongly impact the evolutionary rate **ω** of proteins. Strong Hsp90 substrates are also significantly less abundant, which may offer an alternative explanation for their higher rate of non-synonymous mutations (Figure S5D). Indeed, when taking into consideration the contributions of expression levels and disorder, we found that neither the strong, nor the weak Hsp90 substrates evolved at higher **ω** (strong: p = 0.1; weak: p = 0.594; Figure 4D). However, strong and weak substrates evolved at higher **λ**. Notably, while the difference for the strong substrates is not significant (p = 0.20), the weak substrates evolved at significantly higher relative rates of NC substitutions (p = 0.037; Figure 4E). We speculate that strongly dependent substrates may be more aggregation prone and labile, and therefore less accepting of NC mutations. Strong substrates may also already exhaust all catalytic capacity of the chaperone for their normal folding requirements. In contrast, weakly dependent substrates may be able to fold more easily, and for these proteins chaperones may confer excess robustness that can be exploited by evolutionary pathways. Accordingly, the weak substrates were reported to be more thermodynamically stable than the strong substrates [64], which could explain why they can accommodate more destabilizing mutations, consistent with their higher **λ**.

We next examined the substrates of a co-translationally acting chaperone. The Hsp70 SSB is the main ribosome-associated chaperone involved in the *de novo* folding of nascent polypeptides in *S. cerevisiae* (Figure S5C) [65]. Recent work quantified substrates that strongly and weakly associate with SSB co-translationally [65], indicating that Hsp70 preferentially binds long and disordered nascent chains that may have difficulties to fold on their own [65]. Our analysis revealed that strong SSB substrates evolved at significantly lower **ω** (p<10^−16^, Figure 4F), while weak substrates evolved at similar **ω** compared to non-substrates (p = 0.762; Figure 4F). Interestingly, strong substrates also evolve at lower **λ** (p<10^−16^; Figure 4G). In contrast, weak SSB substrates evolved at significantly higher **λ** (p = 0.035; Figure 4G, S5E). Similar to Hsp90, the more stringent Hsp70 substrates are more folding-challenged and are likely to be most sensitive to mutation, which may explain the strong purifying selection on both the rate of non-synonymous as well as NC substitutions. Also similar to Hsp90, weak substrates depend less stringently on chaperone assistance and may benefit more from the chaperone buffering capacity to accommodate additional NC substitutions. The observed difference between co-translationally acting SSB/Hsp70 and the post-translationally acting Hsp90 is that stringent Hsp70 substrates are characterized by both lower **ω** and **λ**, emphasizing the challenge of *de novo* folding in eukaryotes [53].

Of note, Hsp70/SSB substrates are enriched in proteins with long disordered stretches [65]. Thus, the contribution of chaperones to protein evolution is likely two-fold. On the one hand, chaperones manage the disordered proteome, likely preventing aggregation, thus promoting protein evolution indirectly by potentiating disordered regions. On the other hand, chaperones confer extra robustness to clients, especially to proteins that do not stringently depend on their assistance, and in this way directly promote their evolution. We also examined the substrates of another cotranslationally acting chaperone, the ATP-independent NAC complex, which bind to virtually all nascent chains as they emerge from the ribosome [66]. NAC does not significantly modify either **λ** or **ω**, highlighting that not all chaperones equally promote protein evolution. Our analyses underscore the power of **λ** to disentangle the influence of chaperones on proteome evolution.

### Cost-benefit trade-off in the evolution of protein interaction networks

**Figure 5:**
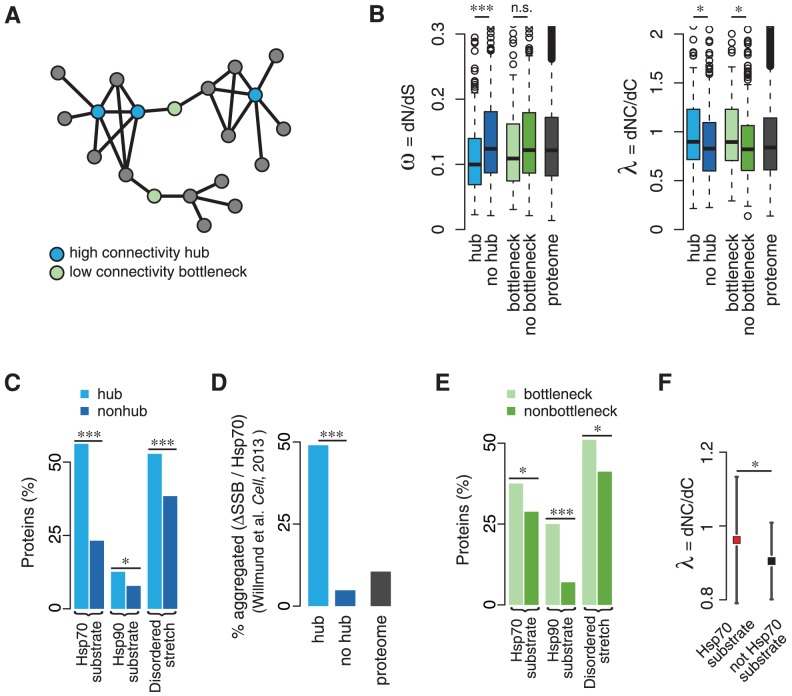
Selective protein quality control in the evolution of protein networks. **A** Hubs comprise the most connected proteins in protein networks, while low connectivity bottlenecks are functionally important network nodes that connect network modules. **B** Hubs and bottlenecks as the most critical proteins in protein interaction networks are characterized by low **ω**, thus strong purifying selective pressure. Despite their generally high degree of conservation, both hubs and bottlenecks exhibit significantly higher distributions of **λ**. **C** Hubs compared to non-hubs are significantly enriched in chaperone substrates and proteins with long disordered stretches. **D** Deletion of the Hsp70s SSB1/2 (data obtained from [65]) results in 50% of the hubs, but less than 10% of the non-hubs to immediately aggregate. While SSB might not directly promote the evolution of hubs, it is instrumental in the homeostasis of intrinsically disordered hub proteins, thus serving as evolutionary potentiator. **E** Bottlenecks are significantly enriched in chaperone substrates and proteins with long disordered stretches compared to non-bottlenecks. **F** The Hsp70 SSB directly promotes non-conservative mutations in bottlenecks in addition to, but independent of intrinsic disorder. Significance levels are indicated as n.s. (not significant), * (p<0.05), and *** (p<0.001).

While the plasticity in evolving novel protein interactions presents a clear evolutionary advantage, the maintenance of intrinsically disordered proteins and chaperone assisted protein folding are energetically costly for the cell [67]. Most chaperones catalyze protein folding in an energy dependent manner, and the targeted degradation and rapid turnover of intrinsically disordered proteins comes at the expense of both increased proteolysis and biosynthesis. This poses the question of the cost-benefit trade-off of promoting NC mutations in the broader context of protein interaction networks. To extend our analysis beyond highly connected proteins, we analyzed the evolution of the most critical proteins in networks, hubs and low-connectivity bottlenecks (Figure 5A) [50]. Hubs are the most highly connected proteins, whereas lowly connected bottlenecks are often key connectors between network modules [68]. Given their functional prominence, they likely evolve under distinct selective pressures. Strikingly, both hubs and bottlenecks evolve at lower or similar **ω** compared to non-hub and non-bottleneck proteins respectively (Wilcoxon rank-sum test; hubs: p = 0.0001, SMD = 0.38; bottlenecks: p = 0.12, SMD = 0.04; Figure 5B). In contrast, both hubs and bottlenecks display significantly higher distributions of **λ** compared to their counterparts (Wilcoxon rank-sum test; hubs: p = 0.04, SMD = 0.24; bottlenecks: p = 0.014, SMD = 0.31; Figure 5B). Of note, highly connected proteins that are components of large and very conserved macromolecular assemblies, such as the ribosome, provide an exception to this trend (Figure S6A,B). The lower relative evolutionary rate **ω** of hubs and bottlenecks can be rationalized given their prevalent high expression levels and functional constraints [50] [68]. Highly connected hubs with several hydrophobic interfaces have to maintain protein solubility, thus reducing the overall number of accepted amino acid changes. On the other hand, those amino acid substitutions that become fixed in hubs and bottlenecks but not in non-hubs and non-bottlenecks appear to be biased towards NC substitutions, likely serving to maintain plasticity to evolve novel interactions.

Our analysis indicates that the evolution of protein networks relies on NC changes in otherwise conserved proteins. This raises the question regarding the mechanisms that protect these proteins from destabilizing and deleterious effects. Remarkably, both hubs compared to non-hubs and bottlenecks compared to non-bottlenecks are more likely to be substrates of Hsp90 and Hsp70, and contain long intrinsically disordered regions (Figure 5C). The strongest determinants of **λ** between hubs and non-hubs are disorder and expression (Figure S7A). We find that hubs are more disordered [69], more highly expressed, and longer lived (Figure S7B). This finding, observed for hubs of protein networks, deviates from the genome-wide trend whereby more disordered proteins are usually less expressed, and turned-over more rapidly [35]. This incongruity led us to examine whether chaperones provide an additional layer of regulation for this subset of unstructured proteins. Indeed, the Hsp70/SSB stabilizes disordered proteins that are highly connected in protein networks, which can explain this discrepancy [65]. Upon deletion of SSB, 50% of the hub proteins, but less than 10% of the non-hubs aggregate immediately (Fisher's exact test: p<10^−7^, Figure 5D) [65]. Our analysis of bottlenecks indicated that these proteins are also preferentially intrinsically disordered and chaperone substrates (Figure 5E). In contrast to hubs, we find that Hsp70/SSB directly promotes NC substitutions in bottlenecks (Regression analysis: p = 0.033, Figure 5F, S7C). However, bottlenecks are also short-lived and turned-over more rapidly (Figure S7D), reflecting their functional role in the dynamic coordination of protein network modules.

**Figure 6:**
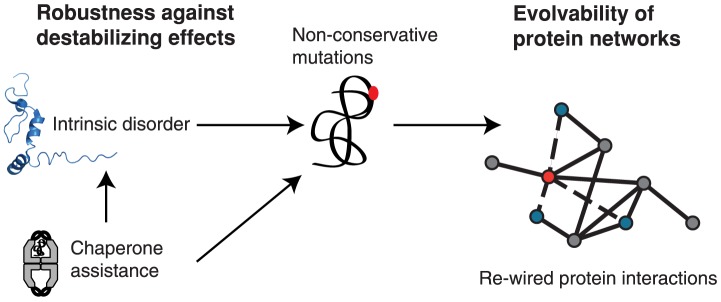
Cost-benefit trade-off in protein network evolvability. Complementary cellular quality control strategies promote non-conservative mutations, thus the evolvability of protein interactions. Intrinsically disordered proteins allow more non-conservative substitutions, but are subject to a more costly regulated turn-over to prevent their aggregation. While energetically expensive, molecular chaperones can promote non-conservative substitutions directly by buffering their destabilizing effect, or indirectly by stabilizing intrinsically disordered proteins. By conferring robustness to otherwise deleterious mutations, protein quality control mechanisms facilitate a higher number of non-conservative mutations, which increases the likelihood of evolving new protein interactions and functions.

We conclude that the energy expenditure of engaging cellular protein homeostasis, i.e. buffering of NC mutations by molecular chaperones, stabilizing IDPs through chaperones or preventing their aggregation through regulated protein turnover, benefits the evolution of protein-protein interaction networks (Figure 6). The associated energetic cost appears to directly trade-off with the re-wiring of protein interactions in critical nodes of protein networks. Importantly, only an integrated analysis can reveal the complex interplay between cellular protein homeostasis and evolution at the proteome level.

## Discussion

To better understand *how* proteins evolve in the cellular context, including the role of chaperones and selective protein quality control on protein evolution at a global proteome level, we incorporated the stability effect of mutations into an extended evolutionary model by defining an evolutionary parameter **λ** that deconvolves the non-synonymous substitutions in proteins into non-conservative (NC) and conservative (C) amino acid changes. Because NC substitutions are more likely to affect protein stability, which is a major biophysical constraint on function [70], **λ** allows to characterize several aspects of protein evolution that are inaccessible to the established evolutionary rate ratio **ω**. In support of our rationale, we found that **λ** can quantify known instances of rapid protein evolution. For instance, **λ** captures the distinct evolutionary patterns of intrinsically disordered proteins, which paradoxically **ω** does not detect. Of note, not all disordered regions are equally variable, and distinct functional roles have been attributed to conserved and flexible disorder [71]. We feel **λ** will become a useful tool to examine the evolution of proteins. The classification used here may be adapted and optimized using additional criteria to improve its usefulness to different problems. Thus, while some non-synonymous mutations will always be non-conservative and destabilizing, there may be species or compartment-specific aspects to consider in the classification of NC vs. C. Further refinements of the concept and implementation of **λ** may help capture specific aspects of protein evolution, for instance within specific organisms or organelles or even within classes of proteins, such as IDPs.

We found the same set of NC substitutions to critically stand out in several independent paradigms: chaperone substrates and intrinsically disordered proteins show elevated rates of NC amino acid changes, and NC substitutions were strongly enriched in proteins that have evolved novel interactions as evident by both the comparison of large-scale protein interaction networks as well as an interaction propensity scale derived from a curated database of protein complexes. Thus, using **λ** we could capture the role of chaperones in conferring robustness against NC mutations, as well as the role of NC mutations in re-wiring of protein networks. Our findings that proteins most critical in protein interaction networks are both highly re-wired despite strong purifying selection, and preferentially supported by chaperones, suggests a general role of chaperones in protein network evolution. Proteins have tightly co-evolved with their cellular environment. By using well-curated sets of chaperone substrates, we find that weak chaperone dependence more strongly promotes protein evolution. Likely, stringently dependent substrates might already have exhausted all chaperone capacity for their normal folding requirements. The evolutionary advantage of weak chaperone dependence thus might also explain the low specificity observed in large and heterogeneous substrates sets for the Hsp70 and Hsp90 chaperone systems. The detailed differentiation between strong vs. weak substrate dependence demonstrates the power of **λ** to analyze this relationship at proteome level. The global link between the evolution of novel protein functions arising from NC mutations, and the buffering role of chaperones is in remarkable agreement with the analysis of accepted mutations from a directed enzyme evolution experiment focused on the bacterial chaperone GroEL [6]. Taken together, these results extend previous findings on the roles of chaperones in protein evolution including observations that they can act as genotypic and phenotypic capacitors [54], [55], [56], buffer the destabilizing effect of mutations upon over-expression [6], facilitate the divergence of gene duplicates [72], and promote greater sequence variability [59].

Our results also fit well in the wider context of related work and theoretical considerations that mutational robustness is central to evolution and adaptation [73]. Protein complex formation itself has been found to stabilize the interaction partners, thus facilitating the evolution of protein networks [74]. In contrast, strong genetic drift alone has been suggested to promote interactome complexity [75], but chaperones may be directly involved in facilitating genetic drift [59]. The finding that proteins that act as phenotypic capacitors, providing robustness against environmental as opposed to genetic perturbations, are also strongly enriched in network hubs [76] indicates a link between environmental and genetic buffering that merits further investigation.

A detailed and mechanistic understanding of protein and proteome evolution will ultimately require contributions from many different approaches at different levels of resolution. Sequence-based evolutionary models will continue providing important insights and assistance in interpreting the increasing amounts of available sequencing data, offering a very efficient indication of distinct selective pressures. Cases of particular interest can be further analyzed with specialized algorithms to predict phenotypic consequences of individual mutations that may achieve higher resolution, especially in well-curated protein families and with available structural information. While in this work the definition of NC and C substitutions is based on predicted stability effects of mutations across the *S. cerevisiae* proteome, species–optimized metrics may increase the strength and significance of insight gained from **λ** in other organisms. The current classification into NC and C substitutions may become less accurate with increasing evolutionary distance from *S. cerevisiae*, raising the question of how **λ** changes with evolutionary distance. Similarly, optimized classifications may further increase the power of **λ** in the analysis of specific sets of proteins, such as IDPs or distinct sub-cellular localizations. To this end, we expect clear advances from novel targeted evolutionary approaches that more explicitly take aspects of protein biophysics and cellular processes into account. New technologies such as deep mutational scanning [77] present an exciting outlook for the experimental characterization in high-throughput of evolutionary constraints on proteins, as well as will provide the necessary data for more accurate classifications of NC and C substitutions independent of potential biases in prediction algorithms, and further improving models of protein evolution.

Cost-benefit trade-offs are ubiquitous in organismal evolution. Our evolutionary parameter **λ** allowed us an integrated analysis that revealed a finely tuned interplay of costly protein homeostasis and beneficial evolution at proteome level. The direct relationship between chaperone assistance and the evolvability of protein networks is intriguing, and may explain the link between expanding chaperone systems with increasing proteome size and complexity [78]. Our work illustrates the importance of integrated as well as more specific and targeted approaches for understanding protein evolution. The increasing availability of biological sequence data provides unique opportunities to incorporate biophysical aspects of protein folding and function into extended evolutionary models, and decipher the cellular contributions to robustness [79]. Our results highlight the importance of considering the cellular context, and in particular the role of molecular chaperones, for understanding protein sequence evolution to achieve a much-needed integrated understanding of protein and proteome evolution.

## Methods

### Computation of evolutionary rates

Protein evolutionary rates and parameters were estimated by maximum likelihood from a Markov model of codon substitutions [9], fitted to pairs of aligned orthologous coding sequences. Because of a shared common ancestor, thus time of divergence, evolutionary distances between pairs of orthologous sequences of closely related species can be interpreted as relative evolutionary rates. Our evolutionary model is described by the rate matrix 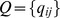, where *q_ij_* is the instantaneous rate from codon *i* to *j* (*i≠j*),
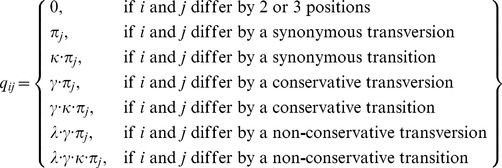



The transition probability matrix 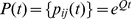 is obtained by maximum likelihood estimation of the parameters *t*, **κ**, **γ**, and **λ** from pairs of aligned orthologous coding sequences by minimizing the log likelihood function
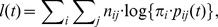
where *n_ij_* is the number of sites, and **π** *_i_* is set to the average genome codon composition of the species compared, in analogy to the F61 model. *t* is the relative divergence time estimated from the relative distance of two orthologous input sequences. **κ** is the transversion-transition ration of all substitutions in the input sequences. **λ** is the ratio of the rates of non-conservative and conservative substitutions. Thus, **λ** indicates the relative partitioning between on average more and less likely destabilizing mutations given all amino acid changes. All calculations were implemented in Matlab, and the function *fminsearch* was used for maximum likelihood estimation. The conventional evolutionary rates *dN* and *dS*, and their ratio **ω** = *dN/dS* were computed with the Matlab function *dndsml*. An independent evolutionary count model based on the established approach by Nei and Gojobori [80] was employed to verify our results (Figure S3A,B).

Generally, the calculation of evolutionary rates is based on counting the number of synonymous, non-synonymous, and here also conservative and non-conservative mutations between aligned orthologs relative to the number of synonymous, non-synonymous, conservative and non-conservative sites in the two sequences, while correcting for multiple substitutions. The maximum likelihood approach explicitly models codon sequence evolution as a stochastic process, and estimates all critical parameters, including the transition/transversion ratio, simultaneously in a probabilistic manner from the sequences. The power of maximum likelihood approaches decreases for small datasets, thus maximum likelihood methods are often applied to larger phylogenies. The count model does not explicitly incorporate transition/transversion biases, but includes mutations that are more than one nucleotide substitution apart by averaging over all possible mutational pathways, and is more robust for small datasets, e.g. low counts of non-conservative mutations. Both approaches produce very similar results (Figure S3A,B).

### Definition of C and NC mutations

Protein stability is a major constraint on protein evolution. The stability effect of mutations depends on the local and global protein fold. However, sequence-based evolutionary models require a clear classification of mutations. In addition, detailed evolutionary trajectories are not known from comparing pair-wise sequence alignments of orthologous sequences, i.e. it is generally not known which sequence has incurred the mutation. We thus chose a simple classification into more likely destabilizing (non-conservative, NC) and less likely destabilizing (conservative C) substitutions to approximate the stability effect of mutations and integrate it into sequence-based evolutionary models. The effect of mutations on protein stability was predicted from the amino acid sequences with the I-Mutant3.0 algorithm [30] for a representative set of ca. 3,000,000 mutations, reflecting 10% of all possible mutations for each protein and randomly sampled across the cytosolic *S. cerevisiae* proteome. This algorithm predicts the effect of mutations with support vector machines that have been trained on experimental data on protein stability. Predictions of stability effects of mutations achieve an accuracy of about 70%. We ranked all amino acid pairs, combined in both directions (i.e *Ala* to *Val* and *Val* to *Ala*), based on how likely there were highly destabilizing (**ΔΔ**G<−2 kcal/mol). Of note, some mutations exhibit clear anisotropy in their mutational effects. For instance, while *Ala* to *Val* mutations is mostly neutral, *Val* to *Ala* mutations are very often predicted to be highly destabilizing. *Val* residues are often found in the tightly packed hydrophobic core of proteins, thus more sensitive to substitutions. Because the direction of the mutation is not normally known, we considered the combined effect between amino acid pairs. Mutations that were in less than 20% of their occurrences highly destabilizing were considered conservative (C), and non-conservative (NC) otherwise. We validated that this is in full agreement with the widely used Blosum62 amino acid substitution matrix in that no NC mutation had a positive Blosum62 score. The conservative and non-conservative substitutions used in this analysis are listed in Table 1. The limited accuracy of the stability predictions, and currently available data to validate them should be kept in mind.

**Table 1:** Conservative and non-conservative amino acid substitutions.

	Conservative	Non-conservative
A	D, E, G, S, T	P, V
C	G, R, S, W, Y	F
D	A, E, G, H, N, V, Y	
E	A, D, G, K, Q, V	
F	I, L, Y	C, S, V
G	A, C, D, E, R	S, V, W
H	D, L, N, P, Q, R, Y	
I	F, L, M, N, V	K, R, S, T
K	E, M, N, Q, R, T	I
L	F, H, I, M, P, Q, R, V, W	S
M	I, K, L, R, T, V	
N	D, H, I, K, S, T, Y	
P	H, L, Q, R, S	A, T
Q	E, H, K, L, P, R	
R	C, G, H, K, L, M, P, Q, T, W	I, S
S	A, C, N, P, T, W, Y	F, G, I, L, R
T	A, K, M, N, R, S	I, P
V	D, E, I, L, M,	A, F, G
W	C, L, R, S	G
Y	C, D, F, H, N, S	

Conservative and non-conservative substitutions used for the computation of **λ**. Only amino acid mutations that are direct neighbors in the genetic code are considered in this analysis. A less stringend definition where more substitutions are classified as non-conservative despite being less often predicted to be destabilizing, e.g. to validate that this classification does not introduce systematic amino acid biases, preserves all observed trends, albeit with less statistical significance.

### Data sources, curation and validation

For our analysis of **λ** in *S. cerevisiae*, we started from 5025 known pairs of orthologous sequences between *S. cerevisiae* and *S. paradoxus*. We removed all alignments of pairs of orthologs if more than 1/3 of the positions in the alignments contained gaps, and required satisfactory convergence of the maximum likelihood in the computation of evolutionary rates (*l_max_*<−1000). This lead to a curated dataset of 3495 pairs of orthologous sequences. Parameter estimation by maximum likelihood becomes inaccurate at very small sample sizes. To test for the robustness of our results, we analyzed protein evolutionary rates for proteins with at least 3 NC substitutions as estimated by maximum likelihood, and validated our findings by evolutionary rates computed by both the maximum likelihood and the count method of all sequences. Orthologs of *E. coli* and *S. salmonella* have diverged less, and we report the evolutionary rates from the count method. Similarly, orthologs of *H. sapiens* and *M. musculus* are more distant, and we report the evolutionary rates calculated by the count method, because these include also mutations that are more than one nucleotide substitution apart. Of note, the parameters from the maximum likelihood and count models are very highly correlated (Figure S3A,B). Genomic sequences and ortholog assignments for *S. cerevisiae* (Scer) and *S. paradoxus* (Spar) were retrieved from the Broad Institute (http://www.broadinstitute.org/regev/orthogroups). We also computed evolutionary rates from aligned orthologs of *E. coli* and *S. salmonella*, and of *H. sapiens* and *M. musculus*, which were obtained from the Ensembl database (http://ensembl.org/biomart/martview/). For orthologs of the more distant *H. sapiens* and *M. Musculus* we required at least 80 aligned codons as described in [14]. Alignments of the genetic sequences between orthologs were computed with ClustalW *via* the corresponding amino acid sequences. Protein abundances for *S. cerevisiae* were obtained from [81], and cellular half-lives from [82]. *S. cerevisiae* mRNA expression levels were taken from [83]. Protein abundances for *E. coli* were retrieved from [84], and the *H. sapiens* “whole organism integrated dataset” from the paxdb database (www.pax-db.org). Intrinsic disorder was predicted with Disopred2 [85]. Classes of structuredness were defined based on the fraction of residues in a protein that was predicted to be disordered, and follows the definition reported in [35]: S (0–10% disordered), M (10–30% disordered), and U (>30% disordered). Local sequence conservation and variability was computed with the program Rate4Site [41]. The GroEL-overexpression dependent mutations were extracted from [6], GroEL substrates from [60], Hsp70 substrates as well as protein aggregates upon Hsp70 deletion from [65], and Hsp90 substrates in *S. cerevisiae* from [86], and in *H. sapiens* from [64]. We pooled class II and III GroEL substrates to achieve a larger sample size. Because GroEL, SSB, and Hsp90 are cytosolic chaperones, we restricted the non-substrate control groups to the cytosolic proteomes of *E. coli*, *S. cerevisiae*, and *H. sapiens* respectively.

The protein-protein interaction networks for *S. cerevisiae* and *S. pombe* were downloaded from the Biogrid database (http://thebiogrid.org). To circumvent a systematic bias due to the very different coverage of the two networks, we extracted a consensus list of highly re-wired proteins as the consensus top 10% proteins in each network with the most “non-orthologous” interactions, i.e. interactions to proteins that do not have orthologs in the other species. We chose this procedure as a very conservative approach to infer re-wired protein interactions. If both binding partners have orthologous proteins in both species, but the exact edge is not present and both networks have very different depth and coverage, it is difficult to systematically know if this is because the edge is in fact not conserved, or simply because the edge was not detected in the much smaller network. In contrast, interactions to proteins that do not have an ortholog in the other organism must be species-specific, thus re-wired. Hubs are the top 10% most highly connected proteins in our study, and non-hubs the bottom 10% connected proteins. Bottlenecks are critical proteins that show a high betweenness centrality, i.e. concentrate the highest density of shortest paths [68]. In this study we only considered proteins as bottlenecks if they were in the top 10% of proteins with the highest betweenness centrality but not also hubs, and non-bottlenecks if they were in the bottom 10% of betweenness centrality.

### Statistical testing

To systematically evaluate the contribution of chaperone assistance to protein evolutionary rates under consideration of confounding factors, we employed non-parametric regression analyses based on a kernel-smoothing approach that has explicitly been developed to test for the significance of categorical predictor variables. The test is fully data driven and robust to over-fitting through extensive cross-validation for smoothing parameter selection and bootstrapping to obtain the null distribution. We used this non-parametric regression model to predict **λ** and **ω** respectively by chaperone dependence, intrinsic disorder, and expression level. Predictor variables that did not improve the overall regression fit were excluded. The regressions of **λ** generally only relied on the input of chaperone dependence and disorder; we found expression to not explain **λ**, consistent with the lack of correlation between **λ** and expression levels. Correlation coefficients of regression fits ranged between R = 0.32 and R = 0.45. Partial regression plots indicate the individual contributions of predictor variables. Error bars were derived from predicting **λ** and **ω** respectively only from individual predictor variables. The relatively large error bars for the contributions of chaperone dependence reflect the limited power of predicting a continuous variable (**λ** or **ω**) only from a categorical one (chaperone substrate or not). This statistical approach is available from the freely available R package “NP” [87].

Differences between distributions of evolutionary rates were tested for statistical significance by Wilcoxon rank-sum tests, and significant enrichments of protein categories in hubs and bottlenecks by Fisher's exact test. Next to p-values, we report the standardized mean difference (SMD) as measure of effect sizes between distributions. All statistical analyses were performed in the statistics environment R (www.r-project.org).

## Supporting Information

Figure S1 **Classification of conservative and non-conservative substitutions.** **A** Distributions of the predicted effects on protein stability for mutations between all amino acid pairs that are separated by one nucleotide substitution in the genetic code. **B** Fraction of highly destabilizing mutations for each amino acid pair. **C** Number of NC amino acid pairs when defined by varying minimum thresholds of predicted highly destabilizing mutations of 10% (NC_10_), 20% (NC_20_) and 30% (NC_30_), and compared to mutations that do not have a positive score in the Blosum62 matrix (NC). **D** Mutation rates of NC substitutions for different thresholds, compared to the rate of non-synonymous mutations (N). **E** Differences in the distributions of predicted stabilities for NC and C mutations for different thresholds.
(EPS)

Figure S2 **NC substitutions have more severe phenotypic or fitness effects.** **A** NC mutations are more damaging than C mutations as indicated by significantly higher PolyPhen scores. **B** NC mutations have more severe phenotypic consequences than C mutations as indicated by significantly lower SNPs3D scores. **C** NC mutations exhibit significantly lower experimentally measured fitness values compared to C mutations based on data from [33].
(EPS)

Figure S3 **Data curation and model validation.** **A** The evolutionary parameter **ω** = *dN/dS* as computed by Maximum Likelihood estimation (ML model; Matlab function dndsml), and computed by an independent count model [80] (Matlab function dnds) correlate very highly. Evolutionary rates and parameters are calculated for aligned orthologs of *S. cerevisiae* and *S. paradoxus*. **B** We implemented an independent extended count model to verify the evolutionary parameter **λ** = *dNC/dC*. The high correlation between **λ** computed by the ML or count model validates the overall approach and implementation. **C** The estimation of additional parameters compared to the model implemented by Yang and Nielson [9] does not affect the maximum likelihood. **D** Distributions of the evolutionary rates *dS*, *dN*, *dC*, and *dNC*. **E** Expression levels are the strongest determinant of the protein evolutionary rate *dN* and the rate ratio **ω**. For *S. cerevisiae* mRNA expression levels and **ω** are clearly correlated (Spearman's correlation coefficient R_s_ = −0.29). **F** Expression levels are not correlated to **λ** (R_S_ = 0.04).
(EPS)

Figure S4 **λ but not ω links to intrinsic disorder.** **A** To avoid a consistent expression bias, structured (S; 0–10% of the residues are predicted to be disordered), moderately unstructured (M; 10–30%) and unstructured (U; >30%) proteins were randomly sampled to have the same distributions of mRNA expression levels. We included n = 749 proteins of each category in our analysis (Wilcoxon rank-sum tests; S vs. M: p = 0.93; M vs. U: p = 0.96). **B** Intrinsic disorder (% of disordered residues) does not correlate to **ω** (R_S_ = 0.07), but **C** correlates to **λ** (R_S_ = 0.36).
(EPS)

Figure S5 **Effect of chaperone assistance and disorder on λ.** **A** Substrates and non-substrates of the chaperonin GroEL in *E. coli* are both on average highly structured, but substrates evolve at higher **λ**. **B** Intrinsic disorder plays a much bigger role in the proteome of the eukaryote *S. cerevisiae* than in the prokaryote *E. coli*. **C** Chaperone networks in eukaryotes are more complex, and split more distinctively into co-translationally acting chaperones that facilitate de novo folding, and post-translationally acting chaperones in response to stress. The main co-translationally acting chaperone in the eukaryote *S. cerevisiae* is the ribosome-associated Hsp70 SSB. The main post-translationally acting heat shock protein is Hsp90. **D** Kinases that are strong Hsp90 substrates [64] evolve at significantly higher rates *dN*, but are also significantly less abundant than kinases that are not Hsp90 substrates. **E** Weak SSB substrates evolve at similar **λ** to non-substrates, but are significantly less disordered. As a result, the effect of SSB to promote **λ** in weak substrates cannot be explained by higher levels of intrinsic disorder. Significance levels are indicated as n.s. (not significant), * (p<0.05), and *** (p<0.001).
(EPS)

Figure S6 **Higher λ in network hubs and bottlenecks.** Hubs and bottlenecks as the most critical proteins in protein interaction networks are characterized by low **ω**, thus strong purifying selective pressure. Despite their generally high degree of conservation, both hubs and bottlenecks exhibit a higher **λ**. **A** The top and bottom 5% of proteins with respect to their network degree and betweenness centrality are compared. When considering only the extreme 5%, the statistical significance for **λ** in the smaller dataset is lost compared to the extreme 10%, but the trends are maintained. **B** The top 1% of proteins with the highest network degree (excluding ribosomal proteins) or betweenness centrality are compared to the bottom 5% of proteins with the lowest network degree or betweenness centrality respectively. We retain the bottom 5% because these proteins are already characterized by the minimal degree 1 or betweenness centrality 0. Proteins that are part of highly conserved large macromolecular complexes such as the ribosome or proteasome do not exhibit a higher **λ** despite their high degree in experimentally characterized protein interaction networks. The full proteome is shown for reference.
(EPS)

Figure S7 **Chaperones facilitate higher λ in network hubs and bottlenecks.** **A** Significance of the predictor variables explaining the higher **λ** in hubs compared to non-hubs. Only expression and disorder are significant predictors in the statistical analysis. **B** Hubs are more disordered, but also more highly expressed and longer-lived. Generally, more disordered proteins are usually less expressed and turned-over more rapidly. It appears that the chaperone SSB allows higher disorder and high, stable expression of its substrates compared to genome wide trends. **C** Significance of the predictor variables explaining the higher **λ** in bottlenecks compared to non-bottlenecks. Both disorder and SSB assistance significantly promote **λ**. **D** Bottlenecks are more disordered, but turned over more rapidly than non-bottlenecks. Significance levels are indicated as n.s. (not significant), * (p<0.05), and *** (p<0.001).
(EPS)

Dataset S1 **Extended evolutionary rates.**
(XLSX)
